# Prevalence of primary headache disorders in Fayoum Governorate, Egypt

**DOI:** 10.1186/s10194-015-0569-6

**Published:** 2015-10-05

**Authors:** Naglaa A. El-Sherbiny, Mohamed Masoud, Nevin M. Shalaby, Hatem S. Shehata

**Affiliations:** Public Health, Fayoum University, Faiyum, Egypt; Neurology, Cairo University, Giza, Egypt

**Keywords:** Primary headache, Prevalence, Migraine, Egypt

## Abstract

**Background:**

There is abundance of epidemiological studies of headache in developed and western countries; however, data in developing countries and in Egypt are still lacking. This study aims to detect the prevalence of primary headache disorders in both urban and rural sectors in Fayoum governorate, Egypt.

**Methods:**

A total of 2600 subjects were included using multi-stage stratified systematic random sampling, with respondent rate of 91.3 %. A pre-designed Arabic version, interviewer-administered, pilot tested structured questionnaire was developed according to The International Classification of Headache Disorders, 3rd edition (beta version), and this questionnaire was validated and the strength of agreement in headache diagnosis was good.

**Results:**

The 1-year headache prevalence was 51.4 %, which was more prevalent in urban dwellers. The most common primary headache type was episodic tension type headache (prevalence; 24.5 %), followed by episodic migraine (prevalence; 17.3 %), both types peaked in midlife. Headache disorders were more common in females with exception of cluster headache that showed the expected male dominance. The risk of chronic headache increased more than one fold and half when the participants were females, married, and in those with high education. More than 60 % of our participants did not seek medical advice for their headaches problem; this percentage was higher in rural areas.

**Conclusions:**

Primary headache disorders are common in Egypt; prevalence rate was comparable with western countries with exception of episodic tension headache. Still headache is under-estimated and under-recognized in Egypt and this problem should be targeted by health care providers.

## Background

Migraine and in general headache disorders are recorded among the top ten causes of disability that interfering with activities of daily living [1]. The one-year prevalence is 10–18 % in migraine, and 31–90 % in tension-type headache (TTH) [2–4].

In Egypt, available data about headache epidemiology are scarce with one study reported a prevalence of migraine to be 2800/100.000 for those aged more than 8 years in Al Quseir city, Red Sea Governorate [5].

Headache is under-diagnosed and also under-treated in developing countries [6, 7]. In Egypt; absence of specialized headache centers, under-estimating the “headache disorders” by household members and even by general practitioners, insufficient patients’ educations, and the availability of pain relievers as over-the-counter (OTC) added more for headache under-recognition. These barriers undoubtedly affect headache care.

Another trackless sector is headache chronification, which was not reported in any available study in Egypt. Chronic headache is associated with more severe disability and lower health related quality of life (HRQoL) compared to episodic headaches [8]. Worldwide, studies in western countries reported that chronic headaches affect approximately 3–4 % (range from 0.5 to 7.3 %) of the adult population [9]. The most frequent subtype is chronic migraine; the prevalence of which ranged from 0.2 % to 5.1 % [9, 10]. Chronic headache can predispose to medication-overuse headache (MOH) with over utilizing different symptomatic medications [10, 11]. According to the International Headache Society Diagnostic (ICHD-IIIb) [12], “MOH is headache occurring on 15 or more days per month as a consequence of regular overuse of acute or symptomatic headache medication (on 10 or more, or 15 or more days per month, depending on the medication) for more than 3 months. MOH is a more severe problem with a prevalence of 1–2 % [9], and its prevalence increases steadily with age [13]. Our primary goals were to assess the prevalence, and estimate the magnitude of primary headache disorders, among the Fayoum population aged ≥ 15 years.

## Methods

### Study design

This study was a community-based, cross-sectional observational descriptive survey.

### Study area

It was conducted in Fayoum Governorate, a developing city in Middle Egypt, 100 kilometres (62 miles) southwest of Cairo. The total population of Fayoum is 3.170.150 inhabitants in January, 2015 with 22.5 % urban and 77.5 % rural population according to Central Agency for Public Mobilization and Statistics (CAPMAS, 2015) [14].

### Sampling method

We used multi-stage stratified systematic random sampling to select the study population. *First*, Fayoum governorate was divided into 6 districts: (Fayoum, Etsa, Tamiya, Sinnuris, Youssef Sadiek, Abshoay). We choose Fayoum district because it is the main and biggest district and has characteristics of urban and rural population as it is surrounded by villages. Fayoum district population represents 27.7 % of the total population of the province. In the *second stage*, two regions located around the university were selected and they represent a rural and urban community, named “Manshiyat Abdallah village” (rural area) and “Keman Faries” (urban area). In the *third stage* the main street was selected in the two regions, then go forward on one direction. In the *fourth stage*, the first house was chosen randomly and then every third house. Eligible study participants were all residents in the selected houses who aged ≥15 years and agreed to participate into the study. If family refused to participate in the survey we take the next house family.

### Sample size

A sample size of 2600 was calculated using a special formula based on reported prevalence of headache from previous epidemiological studies, around 50 % (with 95 % confidence interval for true population mean and precision of 2 %). Finally, the sample was increased by 10 % to overcome problem of non-response and missing data. Out of 2600 questionnaires were distributed, only 2375 completed the questionnaire with a respondent rate of 91.3 %. Data collection was done in ten months from January to October, 2014 by face-to-face interviews conducted by two of the authors (N.E) associate professor and (M.M) lecturer and academic guide in the Public Health Department, Fayoum University.

### Study questionnaire

A pre-designed interviewer-administered structured questionnaire was developed after a review of the literature and prepared in English then translated to Arabic, and then back translation was done by a third party who was blinded of the source language version from Faculty of Arts –English Department– Cairo University. The preliminary questionnaire was then pre-tested in a pilot group for its understandability and making sure that the questions are clear and self-explanatory. The questionnaire is composed of two parts; the first part included demographic, personal and medical aspects (age, gender, education, marital status, occupation, and place of living, contraceptives-pills uses, smoking, hypertension and other relevant medical disorders) and a screening question regarding the presence of headache in the last year (1-year prevalence) “Have you had headache during the last year not related to flu, cold or head injury?” as recommended by earlier studies [13], but “fasting” was added to replace “hangover” to be fitted to the culture. The second part of the questionnaire included questions designed to define the nature and assess patterns of the headache according to the ICHD-IIIb [12]. Individuals who reported headaches ≥ 15 days/month were asked for the criteria of MOH.

### Training of interviewers

The interviewers are (N.E, M.M) received training sessions by one of Neurologist authors (H.S), which included headache diagnosis, types, art of history-taking, patients’ interview and how to introduce the research topic and how to apply the questionnaire to capture all data and to receive the frank answers.

### Pilot surveys

The preliminary questionnaire version was pilot tested on 30 adults both in rural and urban areas (60 % females, mean age 43.6 years, range 18–78). This was done to probe for its simplicity, precision in the words, acceptance and appropriateness for the participants’ educational level and to minimize any leading or confusing questions. This group was not considered in the results; and final modifications were done based on the response of such pilot survey.

### Validity and reliability testing

The Neurologists (H.S) and (N.S) performed 3 field visits to reassess a randomly selected sub-sample of 70 subjects from those who had headaches to evaluate the diagnosis of their headache. The randomization was done using a computer-designed method by one of the interviewers (N.E). By the time of this reassessment, the Neurologists were blinded to the participants’ questionnaire response and they used the ICHD-IIIb criteria [12] to make their diagnosis. This validity test was done within 3 months of initial questionnaire completion. This validity testing method was done according to Kukava et al. [15].

### Diagnosis and data analysis

The questionnaires of individuals who reported headaches were analyzed via algorithmic determinants of the headache characteristics (onset, duration, frequency, site, side-unilateral/bilateral, accompaniments, precipitating factors, etc.). This was based on ICHD-IIIb criteria into: migraine, episodic tension type headache (ETTH), chronic migraine (CM), chronic tension type headache (CTTH), cluster headache and unclassified. Participants who reported headache ≥15 days/month with regular overuse for >3 months of one or more acute/symptomatic treatment drugs were diagnosed to have MOH.

### Ethical considerations

This study was designed according to recommendations of HIS and was approved by Neurology Department Review Board in Cairo University. As the illiteracy rate is high in such rural areas, some of participants cannot sign a written consent. But before administering the questionnaires, the interviewers read the consent form to the participants about the objectives of the study, and the confidentiality of their information. All participants had the right not to participate in the study. Those who had headache were informed of a possible solution for their problem.

### Statistical analysis

#### Data Management

Data were collected, coded and analyzed using Statistical Package for Social Science (SPSS) software version 18 (SSPS, Chicago, IL). Simple descriptive analysis in the form of means and standard deviations were calculated for numerical data. The prevalence was expressed in percentage. In addition to descriptive statistics, non-parametric tests (chi-square) were used to find its association with other factors. Multiple logistic regression analysis was used to analyze risk factors associated with chronic headaches. *P* ≤0.05 was considered statistically significant. For the validity testing, questionnaire sensitivity, specificity, positive predictive value (PPV) and negative predictive values (NPV) were calculated with 95 % confidence intervals (CIs). Cohen’s kappa coefficient was used to estimate overall agreement between diagnoses.

## Results

### Socio-demographic data

The participants’ age ranged from 15 to 83 years with a mean age of 32.32 ± 15.53 years with female: male (1.23:1). The urban residency was 53.1 %, which does not match the geographical urban/rural distribution (22.5 %:77.5 %) [14]. The rural participants were mostly younger, females, less educated, having unskilled occupation and with lesser use of hormonal contraception. The socio-demographic profile of the participants is given in (Table 1).

**Table 1:** Socio-demographic characteristics of the participants

	All	Urban	Rural	*P*-value*
	N (%)	N (%)	N (%)	
Total	2375 (100)	1262 (100)	1113 (100)	
Age (year)				0.09
15–35	993 (41.8)	487 (38.6)	506 (45.5)	
+35–55	754 (31.8)	401 (31.8)	353 (31.7)	
+55	628 (26.4)	374 (29.6)	254 (22.8)	
Gender				0.006
Male	1063 (44.8)	599 (47.5)	465 (41.8)	
Female	1312 (55.2)	663 (52.5)	648 (58.2)	
Education				<0.0001
Illiterate	525 (22.1)	181 (14.3)	344 (30.9)	
Primary education	320 (13.5)	134 (12.2)	186 (16.7)	
Secondary education	862 (36.3)	441 (34.9)	421 (37.8)	
High education	668 (28.1)	506 (40.1)	162 (14.6)	
Occupation				<0.001
Professional	327 (13.8)	217 (17.2)	110 (9.9)	
Managerial and technical	248 (10.4)	139 (11)	109 (9.8)	
Skilled (manual and non-manual)	368 (15.5)	101 (8)	267 (23.9)	
Unskilled	591 (24.9)	265 (21)	326 (29.3)	
Student	210 (8.8)	148 (11.7)	62 (5.6)	
Unemployed	631 (26.6)	392 (31.1)	239 (21.5)	
Marriage				0.07
Yes	1182 (49.8)	603 (47.8)	579 (52)	
No	1193 (50.2)	659 (52.2)	534 (48)	
Contraceptive pills/injection				0.002
Yes	299 (12.6)	182 (14.4)	117 (10.5)	
No	593 (25)	209 (16.6)	384 (34.5)	
N/A	1483 (62.4)	871 (69)	612 (55)	

*P values (Chi-square) compared variables between rural and urban participants

### Headache characteristics

The 1-year headache prevalence was 51.4 % (*n* = 1221), which was more prevalent in urban populations [58.6 % (740/1262) in urban vs. 43.2 % (481/1113) in rural]. According to algorithmic flow, 4 characteristics were identified, (1) pain type (nature), (2) attack duration, (3) headache days/month in the last 3 months to assess for episodicity and chronification, and (4) associated symptoms. (Table 2) showed the headache characteristics.

**Table 2:** Headache characteristics of headache participants

N (%)	
Total	1221 (100)
Headache nature	
Pulsating	553 (45.3)
Aching	408 (33.4)
Both pulsating and aching	128 (10.5)
Others	132 (10.8)
Attack duration	
Less than 4 hours	161 (13.2)
4–72 hours	597 (48.9)
More than 72 hours	346 (28.3)
Undetermined	117 (9.6)
Associated symptoms	
Nausea and/or vomiting	365 (29.9)
Phonophobia and/or photophobia	482 (39.5)
Lacrymation/conjunctival injection	28 (2.3)
Not reported	419 (34.3)
Headache days / month (last 3 months)	
Chronic (≥15 days/month)	184 (15.1)
Episodic (<15 days/month)	1037 (84.9)
Medication overuse headache (MOH)	91 (7.5)

Diagnosis of headache was done by two Neurologists (HS) and (NS) separately according to algorithmic flow assessment based on ICHD-III.

### Headache types

**Fig. 1:**
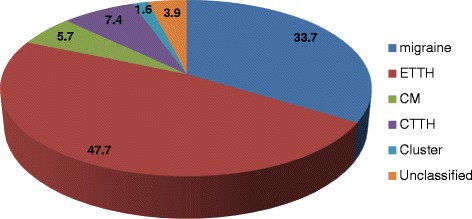
Percentage of headache type diagnoses in headache participants

When the reported diagnoses were not consistent by the two investigators or no definite diagnosis was reached, another combined assessment session was carried out to verify the final diagnosis; however, 3.9 % (48/1221) patients remained unclassified, the percentage of headache type diagnoses in headache participants is shown in (Fig. 1). Headache on ≥15 days/month was reported by 184 participants (15.1 %); about half of them had MOH (91/184).

The observed 1-year prevalence of migraine and ETTH was 17.3 and 24.5 % respectively, with a female preponderance of about 1.8:1 for the former and 1.5:1 for the later. This prevalence of both types peaked in midlife (+35–55 years) and dropped to its lowest level above 55 years. Chronification of headache was more detected in midlife, with female predominance. Male dominance was more in cluster headache with ration (2.2:1). All headache types were more common in urban residency being highest in CTTH (1.9:1) and least in chronic migraine (1.3:1), (Table 3).

**Table 3:** One-year prevalence (% [95 % confidence interval]) of headache types by age, gender and residency

Variables	Migraine	ETTH	CM	CTTH	Cluster	Unclassified
	*N* (%) [CI]	*N* (%) [CI]	*N* (%) [CI]	*N* (%) [CI]	*N* (%) [CI]	*N* (%) [CI]
All	412 (17.3) [15.8–18.8]	583 (24.5) [22.8–26.2]	69 (2.9) [2.2–3.6]	90 (3.8) [3.0–4.6]	19 (0.8) [0.4–1.2]	48 (2.1) [1.5–2.7]
Age (year)						
15–35	133 (32.3) [27.7–36.8]	192 (32.9) [29.1–36.7]	18 (26.1) [15.7–36.5]	24 (26.7) [17.6–35.8]	4 (21.1) [2.8–39.4]	12 (25 %) [12.8–37.3]
+35–55	162 (39.3) [34.5–44]	236 (40.5) [36.5–44.5]	22 (31.9) [20.9–42.9]	28 (31.1) [21.5–40.7]	8 (42.1) [19.9–64.3]	19 (39.6) [25.8–53.4]
+55	117 (28.4) [24–32.7]	155 (26.6) [23–30.2]	29 (42) [30.4–53.6]	38 (42.2) [31.9–52.4]	7 (36.8) [15.1–58.5]	17 (35.4) [21.9–48.9]
Gender						
Male	148 (35.9) [31.3–40.5]	234 (40.1) [36.1–44.1]	24 (34.8) [23.6–46.1]	32 (35.6) [25.7–45.5]	13 (68.4) [47.5–89.3]	19 (39.6) [25.8–53.4]
Female	264 (64.1) [59.5–68.7]	349 (59.9) [55.9–63.9]	45 (65.2) [53.9–76.4]	58 (64.4) [54.5–74.3]	6 (31.6) [10.7–52.5]	29 (60.4) [46.6–74.2]
Residency						
Urban	253 (61.4) [56.7–66.1]	360 (61.7) [57.8–65.6]	39 (56.5) [44.8–68.2]	59 (65.6) [55.8–75.4]	11 (57.9) [35.7–80.1]	18 (37.5) [23.8–51.2]
Rural	159 (38.6 k) [33.9–43.3]	223 (38.3) [34.4–42.2]	30 (43.5) [31.8–55.2]	31 (34.4) [24.6–44.2]	8 (42.1) [19.9–64.3]	30 (62.5) [48.8–76.2]

### Patients’ behavior and headache chronification

Though we had a high response rate (>90 %); yet, surprisingly, we found that 61.7 % (753/1221) did not seek medical advice for their headaches problem. This percentage was higher in rural dwellers (421/753). The main causes were underestimation of their medical problem by patients and/or family members in about half of them (371/753) and the availability of OTC pain relief medications in 25 % (188/753).

Multiple logistic regression analysis for factors associated with chronic headaches revealed that age, female gender, marriage, and education were significantly associated with chronic headache (*p* <0.0001, <0.0001, 0.020, and 0.014 respectively). The risk of chronic headache increased more than one fold and half when the participants were females [OR 1.85–95 % CI (1.49; 2.31)], married [OR 1.56–95 % CI (1.06; 1.89)], and in those with high education and more [OR 1.52–95 % CI (1.09; 2.12)] (Table 4).

**Table 4:** Multiple logistic regressions of factors associated with chronic headache

Factors	Significance	Estimate relative risk (95 % CI)
Age	<0.0001	1.03 (1.02–1.04)
Sex (female vs. male)	<0.0001	1.85 (1.49–2.31)
Marriage (married vs. not married)	0.020	1.41 (1.06–1.89)
Education (high education & more vs. illiterate & low)	0.014	1.52 (1.09–2.12)

### Validity and reliability testing

The results of blind reassessment of a randomly selected 70 sub-sample headache participants are shown in Table 5. Cohen’s Kappa coefficient was 0.75 (95 % C.I; 0.629–0.871), and the strength of agreement in diagnosis of migraine, ETTH, CM and CTTH is considered to be good with high specificity and sensitivity (Table 6).

**Table 5:** Comparison between survey diagnoses and the randomly selected sub-sample blinded diagnoses

Headache type	Survey sample (*n* = 1221)	Sub-sample (*n* = 70)
Migraine	412 (33.7 %)	19 (27.1 %)
ETTH	583 (47.7 %)	26 (37.1 %)
CM	69 (5.7)	9 (12.9 %)
CTTH	90 (7.4 %)	11 (15.7 %)
Cluster headache	19 (1.6 %)	0 (0 %)
Unclassified	48 (3.9 %)	5 (7.2 %)

**Table 6:** Validity testing (sensitivity, specificity, PPV and NPV with 95 % C.I) from the randomly selected headache sub-sample(^a^)

Type of headache	Sensitivity (95 % CI)	Specificity (95 % CI)	PPV (95 % CI)	NPV (95 % CI)
Migraine	0.84 (0.68–1.00)	0.92 (0.85–0.99)	0.80 (0.62–0.98)	0.94 (0.87–1.00)
ETTH	0.85 (0.71–0.98)	0.93 (0.86–1.00)	0.88 (0.75–1.00)	0.91 (0.82–0.99)
CM	0.78 (0.51–1.00)	0.97 (0.92–1.00)	0.78 (0.51–1.00)	0.97 (0.92–1.00)
CTTH	0.91 (0.74–1.00)	0.97 (0.91–1.00)	0.83 (0.62–1.00)	0.98 (0.94–1.00)
Unclassified	0.40 (0.03–0.83)	0.97 (0.92–1.00)	0.50 (0.01–0.99)	0.95 (0.90–1.00)

(^a^)Validity measures cannot be calculated for cluster headache because there were no cases of it in sub-sample results

*PPV* positive predictive value, *NPV* negative predictive value

## Discussion

This study is the first comprehensive population-based survey to assess the prevalence of primary headache disorders, among the Fayoum governorate population in Egypt. The epidemiology of headache in Egypt that was addressed by the other study done in Al Quseir city [5] was a part of the epidemiology of different neurological disorders in Egypt and it only reported the prevalence of migraine.

The points of strength in our study included developing a new structured interviewer-administered questionnaire in Arabic, which was pilot tested and validated. The source language “English” was translated to Arabic language and then back to English by another translator who was blinded to the source language version to evaluate for the quality of translation. In the questionnaire development we were not only concerned with literal translation, but also with words meanings and how they are linked to local realities and culture [16]. Also, we made sure that the developing questionnaire was simple, with precise words and could adequately measure headache types; all these factors were addressed to avoid inconsistency and lack of agreement between interviewers and to ensure questionnaire validity in terms of credibility and better quality data [17] to be used in further research on wider scales in the Arabic speaking countries.

Possible limitations of our study included the cross-sectional design, which does not cover different types of headaches that can occur in the same patient and may require prospective cohort using headache diaries [18]. In addition, the interviewers faced difficulties especially in rural clusters, not only due to insufficient resources and deficient research capabilities, but also due to potential communication barriers between interviewers and participants that included local traditions where people may not open their doors to strangers, the rustic attitude of some participants and difficulty to interview participants privately, especially with female participants.

A considerable point is the higher response rate, which was calculated on the basis of returned questionnaires. This is attributed to familiarity of the interviewers to the studied populations, as they are performing repeated field visits as a part of ‘public health curriculum for undergraduate medical students’ in the past 8 years; also, those people are considering medical visitors as honored guests and offer hospitality to them. However; this was less detected in rural conservative societies.

In the current series, the 1-year headache prevalence was 51.4 %, with 61.5 % of our patients were women, this is similar to the prevalence of headache in Europe, where 53 % of adults have current headache (61 % among women) [2], and slightly higher than the global estimate which was 46 % [9]. In Africa, a higher prevalence was recorded in Zambia (72 %) (gender- and habitation-adjusted 61.6 %) [13]; whereas, lower prevalence was reported in Ethiopea (21.6 %) [19] and Tanzania (23.1 %) [20]. The discrepancy of prevalence could be attributed to different methodologies used, as well as cultural and population characteristics of the studied patients; however, in developing countries the limited funding, larger rural dweller and the lower profile of headache disorders compared with other diseases stand as main obstacles for systemic data collection [19, 21]. The female predominance is almost a consistent finding in many other studies, which reflects the fact that primary headaches are more common in women [6].

In our survey, 58.6 % of our participants were urban dwellers and this does not match the geographical urban/rural distribution (22.5 %:77.5 %), this imbalance in sampling was previously reported by Mbewe et al. [13], and they pointed to the difficulties facing the interviewers in reaching the rural sector. This was also applied in our survey; however, a shortage in financial resources and a higher non-respondent rate in this conservative society added to our challenges. Moreover, in our study all headache types were more common in urban residency being highest in CTTH; this higher prevalence is related to many factors, such as higher psychosocial stressors in urban areas with a more complicated life style in addition to higher educational level; as generally, the prevalence of primary headaches increased and secondary headaches decreased with educational level [6]. In Egypt, rural people often trivialize headache and under-estimate it because of other more demanding and major health problems; also the hard physical labor in rural population can be a factor as it was previously documented that heavy exercises are associated with decreased risk of migraine [22].

The most common headache type in our sample was ETTH (24.5 %), followed by migraine (17.3 %), both types were peaked in mid-life and dropped to its lowest level above 55 years; these findings were similar to what reported in rural population in Cuba (TTH was 25.56 %, migraine was 16.94 %) [23]. However, several studies reported a different prevalence of headache types; again, this could be attributed to different methodologies and cultural diversity [6]. In Eurolight Project [2, 24], the mean prevalence of migraine in Europe was 14.7 %, while the overall prevalence of TTH was 62.6 %. In Georgia, the prevalence was 37.3 % for TTH and 15.6 % for migraine [25]. It is worth noting that prevalence of both headache types peaked in midlife and dropped to its lowest level above 55 years; this somewhat lower prevalence in elderly was previously reported [26].

When it comes to chronic headache, the 1-year prevalence of chronic migraine in our sample was 2.9 %, while CTTH was 3.8 %. The least reported headache type was cluster headache (0.8 %). In their review, Stovner and Andree [2] showed that CTTH occurred in 3.3 %; while the one-year cluster headache prevalence was unknown, yet, its lifetime prevalence was 0.2–0.3 %. Cluster headache (CH) is probably under-diagnosed and unrecognized with a significant diagnostic delay in most patients. Many studies are biased by the sample chosen and by the difficulties of diagnosing CH by mailed questionnaires or telephone interviews. In a tertiary care headache clinic, CH was diagnosed in 2.73 % of patients [27]. The higher prevalence of CH in Egyptian patients may be due to racial, lifestyle and/or cultural factors.

Population-based epidemiological studies showed that chronic headaches affect approximately 3–4 % (range from 0.5 to 7.3 %) of the adults in western countries [9] and ranged from 1 to 4 % in the Asian-Pacific population [28]. The most frequent subtype is chronic migraine (prevalence range; 0.2–5.1 %) [29].

About half of our patients with chronic headache had MOH with prevalence of 3.8 %. It is well-established that medication overuse is frequent among those with chronic headache [2]; in a European survey probable medication overuse headache was found in 3 % [24]; however, lower prevalence rates were reported in Germany (2 %) [30], Norway (1.7 %) [31] and Spain (1 %) [32].

In our series, chronic headache was more in midlife patients with female predominance, moreover, the risk increased with marriage and higher education. Many studies identified number of risk factors for chronic headache, which included age, family history, smoking, obesity, snoring, sleeping problems, head injury, stressful life periods and low educational level [33]. It is well known that development of chronic migraine has been associated with both non-modifiable risk factors (female gender, low socio-economic status and level of schooling) and modifiable risk factors (anxiety, depression, sleep apnea/snoring, obesity and consumption of painkillers) [34]. However, these factors were not addressed at our work and it was beyond the scope of our aim of work.

Outstandingly, 61.7 % of our headache patients did not seek medical advice especially among rural dwellers. This high percentage points to headache under-recognition and under-estimation in low and middle income countries [7]. In Egypt, absence of specialized headache centers, availability of pain relievers as OTC and lack of patients’ education are other major causes.

## Conclusion

In conclusion, primary headache disorders are common in Egypt and the prevalence rates are comparable to western countries regarding migraine and chronic headaches; however, in Egypt a lower prevalence rate was recorded in episodic tension headache; yet, it is still the most common headache type in both urban and rural provinces; with higher prevalence of cluster headache. In Egypt; health care providers have to overcome many obstacles for better headache care, the most important of which is the increase of patients’ awareness of the early symptoms to seek medical help.
